# Supplementary material for: Estimating post-operative complication rates in patients with primary brain tumours from routine administrative data: A national cohort study
Source: PLoS One. 2026 Feb 19;21(2):e0342011. doi: 10.1371/journal.pone.0342011 (PMC12919839; doi:10.1371/journal.pone.0342011)
Supplement: S1 Appendix — (DOCX) [file pone.0342011.s001.docx]

### **S1 Appendix. Data and coding details**

This is a supplementary document to cover in more detail data, coding systems and codes used in our work.

###

**Ethics approval and secure environment**

This project has REC/HRA approval (REC reference: 19/YH/0213).

All work was carried out using secure computing facilities hosted in the Big Data Analysis Unit (BDAU) at Imperial College London.

**Coding systems**

AHRQ PSIs uses ICD-9-CM or ICD-10-CM/PCS.

Procedure codes in the UK are coded using OPCS-4

Diagnosis codes in the UK are coded using ICD-10-WHO, international classification of diseases, 10th revision

**Codes**

DISDEST code ‘79’ used for selecting patients that died in-hospital

VITALSTATUS value of ‘D’, ‘D4’, ‘D5’ used for selecting patients that died within 30-days after surgery

Personal history codes: ICD-10 codes starting with a ‘Z’ e.g. Z81 - Family history of mental and behavioural disorders, Z72.1 - Problems related to lifestyle: alcohol use

Brain/spinal tumour codes excluded from most common post-surgical diagnosis codes list were ICD-10-WHO codes starting with C70, C71, C72;

Brain tumour diagnoses were grouped as follows:

- cranial glioblastoma (ICD-10:C71, ICD-O:9440-9442)
- other cranial tumours (ICD-10:C71+any other morphology than ICD-O:9440-9442 OR ICD-10:C71+ICD-O:9440-9442+Grade:1-2 (those that had a glioblastoma morphology, but grade 1 and 2 and were most likely miscoded other cranial tumours, as indicated by survival curves which were different from other grade glioblastoma patients))
- acoustic neuroma (ICD-10:D33.3)
- cranial meningioma (ICD-10:D32.0, ICD-O:9530)
