# Supplementary material for: Estimating post-operative complication rates in patients with primary brain tumours from routine administrative data: A national cohort study
Source: PLoS One. 2026 Feb 19;21(2):e0342011. doi: 10.1371/journal.pone.0342011 (PMC12919839; doi:10.1371/journal.pone.0342011)
Supplement: S2 Table — (DOCX) [file pone.0342011.s002.docx]

**S2 Table. List of OPCS 4 codes used to define major resection and biopsy**

| **Major resection** | |
| --- | --- |
| **Code** | **Label** |
| A012 | Total lobectomy of brain |
| A013 | Partial lobectomy of brain |
| A018 | Other specified major excision of tissue of brain |
| A019 | Unspecified major excision of tissue of brain |
| A021 | Excision of lesion of tissue of frontal lobe of brain |
| A022 | Excision of lesion of tissue of temporal lobe of brain |
| A023 | Excision of lesion of tissue of parietal lobe of brain |
| A024 | Excision of lesion of tissue of occipital lobe of brain |
| A025 | Excision of lesion of tissue of cerebellum |
| A026 | Excision of lesion of tissue of brain stem |
| A027 | Excision of transcranial dermoid cyst |
| A028 | Other specified excision of lesion of tissue of brain |
| A029 | Unspecified excision of lesion of tissue of brain |
| A073 | Exploration of tissue of brain |
| A078 | Other specified other open operations on tissue of brain |
| A108 | Other specified other operations on tissue of brain |
| A118 | Other specified operations on tissue of brain |
| A168 | Other specified other open operations on ventricle of brain |
| A171 | Endoscopic extirpation of lesion of ventricle of brain |
| A208 | Other specified other operations on ventricle of brain |
| A291 | Excision of lesion of optic nerve (ii) |
| A293 | Excision of lesion of trigeminal nerve (v) |
| A295 | Excision of lesion of acoustic nerve (viii) |
| A298 | Excision of lesion of specified cranial nerve NEC |
| A381 | Extirpation of lesion of meninges of cortex of brain |
| A382 | Extirpation of lesion of meninges of sphenoidal ridge of cranium |
| A383 | Extirpation of lesion of meninges of subfrontal region of brain |
| A384 | Extirpation of lesion of meninges of parasagittal region of brain |
| A385 | Extirpation of lesion of falx cerebri |
| A386 | Extirpation of lesion of tentorium cerebelli |
| A388 | Other specified extirpation of lesion of meninges of brain |
| A389 | Unspecified extirpation of lesion of meninges of brain |
| A428 | Other specified other operations on meninges of brain |
| A431 | Extirpation of lesion of meninges of skull base |
| A432 | Extirpation of lesion of meninges of skull clivus |
| A438 | Other specified other extirpation of lesion of meninges of brain |
| A441 | Chordectomy of spinal cord |
| A442 | Extirpation of lesion of spinal cord NEC |
| A443 | Excision of lesion of intradural intramedullary spinal cord |
| A444 | Excision of lesion of extradural spinal cord |
| A445 | Excision of lesion of intradural extramedullary spinal cord |
| A448 | Other specified partial extirpation of spinal cord |
| A449 | Unspecified partial extirpation of spinal cord |
| A511 | Extirpation of lesion of meninges of spinal cord |
| A518 | Other specified other operations on meninges of spinal cord |
| A571 | Extirpation of lesion of spinal nerve root |
| A599 | Unspecified excision of peripheral nerve |
| A611 | Excision of lesion of peripheral nerve |
| B012 | Trans-sphenoidal hypophysectomy |
| B068 | Other specified operations on pineal gland |
| C021 | Excision of lesion of orbit |
| E158 | Other specified operations on sphenoid sinus |
| T962 | Excision of lesion of soft tissue NEC |
| V031 | Exploratory open craniotomy |
| V038 | Other specified opening of cranium |
| V039 | Unspecified opening of cranium |
| V051 | Extirpation of lesion of cranium |
| V058 | Other specified other operations on cranium |
| V431 | Excision of lesion of cervical vertebra |
| V433 | Excision of lesion of lumbar vertebra |
| V498 | Other specified exploration of spine |
| V499 | Unspecified exploration of spine |
| Y059 | Unspecified excision of organ NOC |
| Y068 | Other specified excision of lesion of organ NOC |
| Y069 | Unspecified excision of lesion of organ NOC |
| Y461 | Trans-sphenoidal open approach to contents of cranium |
| Y463 | Transoral open approach to contents of cranium |
| Y464 | Transmastoid open approach to contents of cranium |
| Y465 | Supratentorial open approach to contents of cranium |
| Y467 | Craniectomy approach to contents of cranium |
| Y468 | Other specified open approach to contents of cranium |
| Y469 | Unspecified open approach to contents of cranium |
| **Biopsy** | |
| **Code** | **Label** |
| A041 | Open biopsy of lesion of tissue of frontal lobe of brain |
| A042 | Open biopsy of lesion of tissue of temporal lobe of brain |
| A043 | Open biopsy of lesion of tissue of parietal lobe of brain |
| A044 | Open biopsy of lesion of tissue of occipital lobe of brain |
| A045 | Open biopsy of lesion of tissue of cerebellum |
| A046 | Open biopsy of lesion of tissue of brain stem |
| A048 | Other specified open biopsy of lesion of tissue of brain |
| A049 | Unspecified open biopsy of lesion of tissue of brain |
| A081 | Biopsy of lesion of tissue of frontal lobe of brain NEC |
| A082 | Biopsy of lesion of tissue of temporal lobe of brain NEC |
| A083 | Biopsy of lesion of tissue of parietal lobe of brain NEC |
| A084 | Biopsy of lesion of tissue of occipital lobe of brain NEC |
| A085 | Biopsy of lesion of tissue of cerebellum NEC |
| A086 | Biopsy of lesion of tissue of brain stem NEC |
| A088 | Other specified other biopsy of lesion of tissue of brain |
| A089 | Unspecified other biopsy of lesion of tissue of brain |
| A104 | Aspiration of lesion of tissue of brain NEC |
| A105 | Puncture of tissue of brain NEC |
| A181 | Diagnostic endoscopic examination of ventricle of brain and biopsy of lesion of ventricle of brain |
| A188 | Other specified diagnostic endoscopic examination of ventricle of brain |
| A363 | Biopsy of lesion of cranial nerve |
| A422 | Biopsy of lesion of meninges of brain |
| A454 | Open biopsy of lesion of spinal cord |
| A456 | Open aspiration of lesion of spinal cord |
| A481 | Biopsy of lesion of spinal cord NEC |
| A482 | Aspiration of lesion of spinal cord |
| A513 | Biopsy of lesion of meninges of spinal cord |
| A578 | Other specified operations on spinal nerve root |
| A731 | Biopsy of lesion of peripheral nerve |
| B042 | Biopsy of lesion of pituitary gland |
| T968 | Other specified other operations on soft tissue |
| V036 | Exploratory burrhole of cranium |
| V052 | Biopsy of lesion of cranium |
| Y201 | Stereotactic biopsy of lesion of organ NOC |
| Y202 | Stereotactic biopsy of organ NOC |
| Y208 | Other specified biopsy of organ NOC |
| Y462 | Frontal open approach to contents of cranium |
| Y466 | Infratentorial open approach to contents of cranium |
| Y471 | Trans-sphenoidal burrhole approach to contents of cranium |
| Y472 | Frontal burrhole approach to contents of cranium |
| Y473 | Transoral burrhole approach to contents of cranium |
| Y474 | Transmastoid burrhole approach to contents of cranium |
| Y475 | Supratentorial burrhole approach to contents of cranium |
| Y476 | Infratentorial burrhole approach to contents of cranium |
| Y478 | Other specified burrhole approach to contents of cranium |
| Y479 | Unspecified burrhole approach to contents of cranium |
| Y698 | Other specified harvest of other tissue |
