# Supplementary material for: Estimating post-operative complication rates in patients with primary brain tumours from routine administrative data: A national cohort study
Source: PLoS One. 2026 Feb 19;21(2):e0342011. doi: 10.1371/journal.pone.0342011 (PMC12919839; doi:10.1371/journal.pone.0342011)
Supplement: S3 Table — Codes were obtained from the OECD Health Care Quality and Outcomes (HCQO) 2018−19 Data Collection: https://www.oecd.org/statistics/data-collection/Health%20Care%20Quality%20Indicators_guidelines.pdf (* - codes excluded from our study). (DOCX) [file pone.0342011.s003.docx]

**S3 Table. OECD Patient safety (PS) questionnaire codes.** Codes were obtained from the OECD Health Care Quality and Outcomes (HCQO) 2018-19 Data Collection: <https://www.oecd.org/statistics/data-collection/Health%20Care%20Quality%20Indicators_guidelines.pdf> (*  - codes excluded from our study)

| **ICD-10 code** | **Diagnosis description** |
| --- | --- |
| ***Retained surgical item or unretrieved device fragment*** | |
| T81.5 | Foreign body accidentally left in body cavity or operation wound following a procedure |
| T81.6 | Acute reaction to foreign substance accidentally left during a procedure |
| Y61.0 | Foreign object accidentally left in body during surgical and medical care: During surgical operation |
| Y61.1 | Foreign object accidentally left in body during surgical and medical care: During infusion or transfusion |
| Y61.2 | Foreign object accidentally left in body during surgical and medical care: During kidney dialysis or other perfusion |
| Y61.3 | Foreign object accidentally left in body during surgical and medical care: During injection or immunization |
| Y61.4 | Foreign object accidentally left in body during surgical and medical care: During endoscopic examination |
| Y61.5 | Foreign object accidentally left in body during surgical and medical care: During heart catheterization |
| Y61.6 | Foreign object accidentally left in body during surgical and medical care: During aspiration, puncture and other catheterization |
| Y61.7 | Foreign object accidentally left in body during surgical and medical care: During removal of catheter or packing |
| Y61.8 | Foreign object accidentally left in body during surgical and medical care: During other surgical and medical care |
| Y61.9 | Foreign object accidentally left in body during surgical and medical care: During unspecified surgical and medical care |
| ***Pulmonary Embolism*** | |
| I26.0 | Pulmonary embolism with mention of acute cor pulmonale |
| I26.9 | Pulmonary embolism without mention of acute cor pulmonale |
| ***Pulmonary Embolism and Deep Vein Thrombosis*** | |
| I80.1 | Phlebitis and thrombophlebitis of femoral vein |
| I80.2 | Phlebitis and thrombophlebitis of other deep vessels of lower extremities |
| I80.3 | Phlebitis and thrombophlebitis of lower extremities, unspecified |
| I80.8 | Phlebitis and thrombophlebitis of other sites |
| I80.9 | Phlebitis and thrombophlebitis of unspecified site |
| I82.8 | Embolism and thrombosis of other specified veins |
| ***Sepsis*** | |
| A40.0 | Septicaemia due to streptococcus, group a |
| A40.1 | Septicaemia due to streptococcus, group b |
| A40.2 | Septicaemia due to streptococcus, group d |
| A40.3 | Septicaemia due to streptococcus pneumoniae |
| A40.8 | Other streptococcal septicaemia |
| A40.9 | Streptococcal septicaemia, unspecified |
| A41.0 | Septicaemia due to staphylococcus aureus |
| A41.1 | Septicaemia due to other specified staphylococcus |
| A41.2 | Septicaemia due to unspecified staphylococcus |
| A41.3 | Septicaemia due to haemophilus influenza |
| A41.4 | Septicaemia due to anaerobes |
| A41.5 | Septicaemia due to other gram-negative organisms |
| A41.8 | Other specified septicaemia |
| A41.9 | Septicaemia, unspecified |
| R57.2 | Septic shock |
| R57.8 | Other shock |
| R65.0 | Systemic Inflammatory Response Syndrome of infectious origin without organ failure |
| R65.1 | Systemic Inflammatory Response Syndrome of infectious origin with organ failure |
| T81.1 | Shock during or resulting from a procedure, not elsewhere classified |
| ***Wound dehiscence*** | |
| T81.3 | disruption of a wound not elsewhere classified |
| ***Obstetric Trauma*** | |
| ***** O70.2 | Third degree perineal laceration during delivery |
| ***** O70.3 | Fourth degree perineal laceration during delivery |
