# Supplementary material for: Estimating post-operative complication rates in patients with primary brain tumours from routine administrative data: A national cohort study
Source: PLoS One. 2026 Feb 19;21(2):e0342011. doi: 10.1371/journal.pone.0342011 (PMC12919839; doi:10.1371/journal.pone.0342011)
Supplement: S4 Table — (DOCX) [file pone.0342011.s004.docx]

**S4 Table. The list of top 100 diagnosis codes extracted from index admission**

| Code | Description |
| --- | --- |
| I10X | Hypertension |
| D320 | Benign neoplasm: Cerebral meninges |
| D352 | Benign neoplasm of pituitary gland |
| F171 | Mental and behavioural disorders due to use of tobacco : harmful use |
| E119 | Type 2 diabetes mellitus. Without complications |
| J459 | Asthma, unspecified |
| E780 | pure hypercholesterolaemia*** |
| F329 | Depressive episode, unspecified |
| E039 | Hypothyroidism, unspecified |
| R568 | Other and unspecified convulsions |
| G936 | cerebral oedema** |
| G409 | Epilepsy, unspecified |
| D333 |  |
| E669 | Obesity, unspecified |
| F419 | Anxiety disorder, unspecified |
| T810 | Haemorrhage and haematoma complicating a procedure, not elsewhere classified (at any site resulting from a procedure) |
| Y838 | Other surgical procedures**** |
| K219 | Gastro-oesophageal reflux disease without oesophagitis |
| Y836 | Removal of other organ (partial) (total) |
| D321 |  |
| G938 | Other specified disorders of brain |
| N390 | Urinary tract infection, site not specified |
| G819 | Hemiplegia, unspecified |
| R470 | Dysphasia and aphasia |
| D361 |  |
| J449 | Chronic obstructive pulmonary disease, unspecified |
| H534 | Visual field defects |
| I259 | Chronic ischaemic heart disease, unspecified |
| M199 | Arthrosis, unspecified |
| G960 | Cerebrospinal fluid leak |
| H919 | Hearing loss, unspecified |
| G919 | Hydrocephalus, unspecified |
| I252 | Old myocardial infarction |
| I48X | Atrial fibrillation and flutter |
| E871 | Hypo-osmolality and hyponatraemia |
| N40X | Hyperplasia of prostate |
| G935 | compression of brain |
| G473 | sleep apnoea |
| R33X | Retention of urine |
| G941 | Hydrocephalus in neoplastic disease |
| K449 | Diaphragmatic hernia without obstruction or gangrene |
| E220 | Acromegaly and pituitary gigantism |
| I489 | Atrial fibrillation and atrial flutter, unspecified |
| K590 | Constipation |
| I209 | Angina pectoris, unspecified |
| R296 | Tendency to fall, not elsewhere classified |
| G992 | Myelopathy in diseases classified elsewhere |
| G439 | Migraine, unspecified |
| D329 |  |
| M139 | Arthritis, unspecified |
| R410 | Disorientation |
| Y95X | Nosocomial condition |
| R298 | Other and unspecified symptoms and signs involving the nervous and musculoskeletal systems |
| R51X | Headache |
| R11X | Nausea and vomiting |
| M109 | Gout, unspecified |
| K589 | Irritable bowel syndrome NOS |
| E230 | Hypopituitarism |
| M819 | Osteoporosis, unspecified |
| G510 | Bell palsy |
| D430 |  |
| M069 | Rheumatoid arthritis, unspecified |
| J181 | Lobar pneumonia, unspecified |
| G403 | Generalized idiopathic epilepsy and epileptic syndromes |
| D649 | Anaemia, unspecified |
| R900 | Intracranial space-occupying lesion |
| R268 | Other and unspecified abnormalities of gait and mobility |
| I959 | Hypotension, unspecified |
| G911 | Obstructive hydrocephalus |
| F101 | Mental and behavioural disorders due to use of alcohol : harmful use |
| H409 | Glaucoma, unspecified |
| I269 | Pulmonary embolism without mention of acute cor pulmonale |
| G978 | Other postprocedural disorders of nervous system |
| I639 | Cerebral infarction, unspecified |
| R13X | Dysphagia |
| H532 | Double vision |
| K579 | Diverticular disease of intestine NOS |
| R001 | Brachycardia |
| T812 | Accidental punctue or laceration during procedure |
| N179 | Acute renal failure, unspecified |
| D432 |  |
| M179 | Gonarthrosis, unspecified |
| J189 | Pneumonia, unspecified |
| E232 | Diabetes insipidus |
| B962 | Escherichia coli [E. coli] as the cause of diseases classified to other chapters |
| J22X | Unspecified acute lower respiratory infection |
| D180 | heamangioma, any site |
| N183 | Chronic kidney disease, stage 3 |
| H538 | other visual disturbances |
| D334 |  |
| Y600 | Unintentional cut, puncture, perforation or haemorrhage during surgical and medical care: during surigical operation |
| G401 | Localization related partial symptomatic epilepsy and epileptic syndromes |
| E668 | other obesity |
| M797 | Fibromyalgia |
| I678 | Other specified cerebrovascular diseases |
| T814 | infection following a procedure, not elsewhere classified* |
| G939 | disroder of brian, unspecified |
| H931 | tinnitus |
| R32X | Unspecified urinary incontinence |
| L409 | psoriasis |
