# Supplementary material for: Estimating post-operative complication rates in patients with primary brain tumours from routine administrative data: A national cohort study
Source: PLoS One. 2026 Feb 19;21(2):e0342011. doi: 10.1371/journal.pone.0342011 (PMC12919839; doi:10.1371/journal.pone.0342011)
Supplement: S5 Fig — (PDF) [file pone.0342011.s005.pdf]

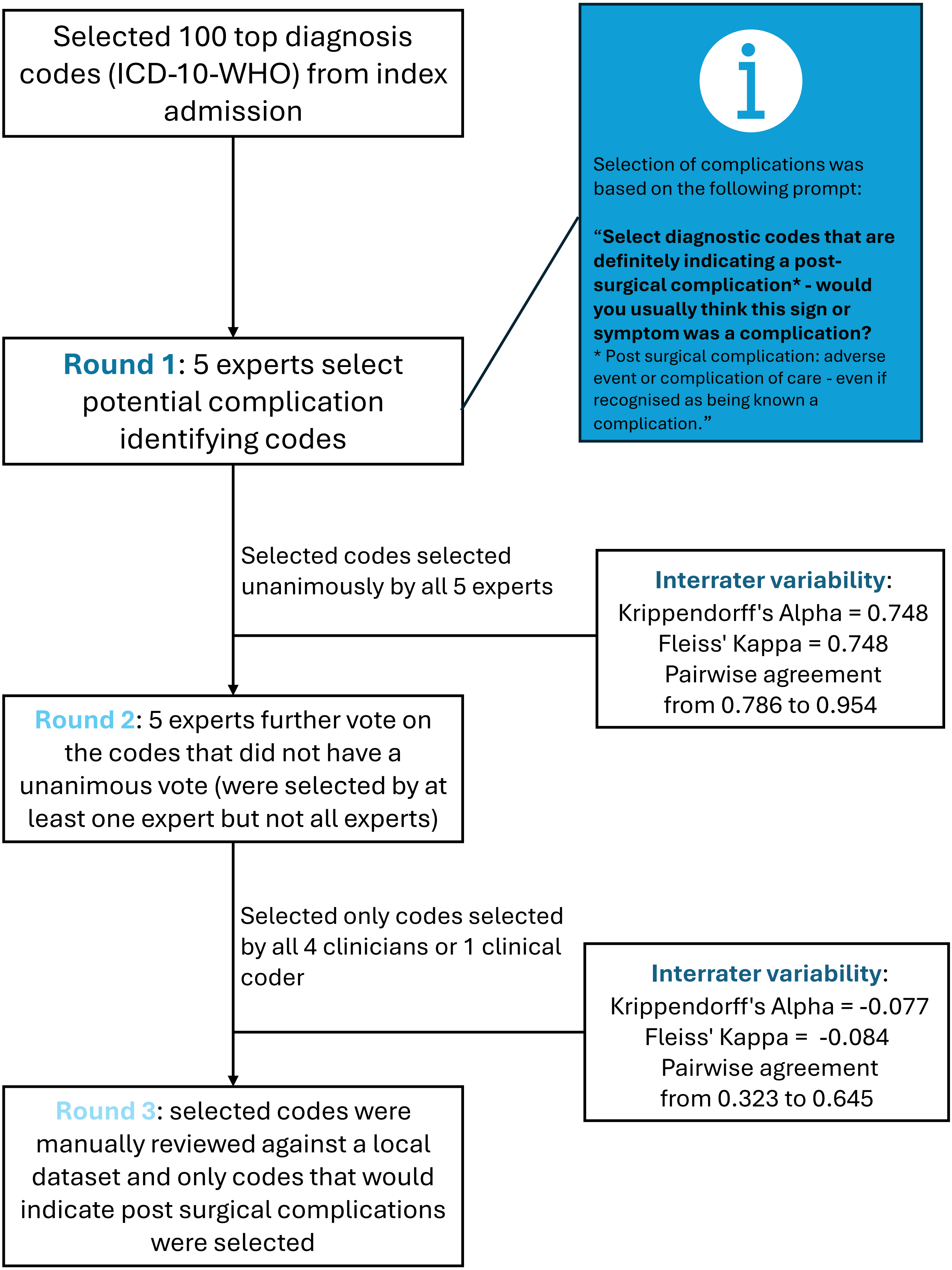
Selected 100 top diagnosis codes (ICD-10-WHO) from index admission
Round 1: 5 experts select potential complication identifying codes
Selection of complications was based on the following prompt:
“Select diagnostic codes that are definitely indicating a post-surgical complication* - would you usually think this sign or symptom was a complication?
* Post surgical complication: adverse event or complication of care - even if recognised as being known a complication.”
Selected codes selected unanimously by all 5 experts
Interrater variability:
Krippendorff's Alpha = 0.748
Fleiss' Kappa = 0.748
Pairwise agreement from 0.786 to 0.954
Round 2: 5 experts further vote on the codes that did not have a unanimous vote (were selected by at least one expert but not all experts)
Selected only codes selected by all 4 clinicians or 1 clinical coder
Interrater variability:
Krippendorff's Alpha = -0.077
Fleiss' Kappa = -0.084
Pairwise agreement from 0.323 to 0.645
Round 3: selected codes were manually reviewed against a local dataset and only codes that would indicate post surgical complications were selected
