# Supplementary figures and images for: Estimating post-operative complication rates in patients with primary brain tumours from routine administrative data: A national cohort study
Source: PLoS One. 2026 Feb 19;21(2):e0342011. doi: 10.1371/journal.pone.0342011 (PMC12919839; doi:10.1371/journal.pone.0342011)

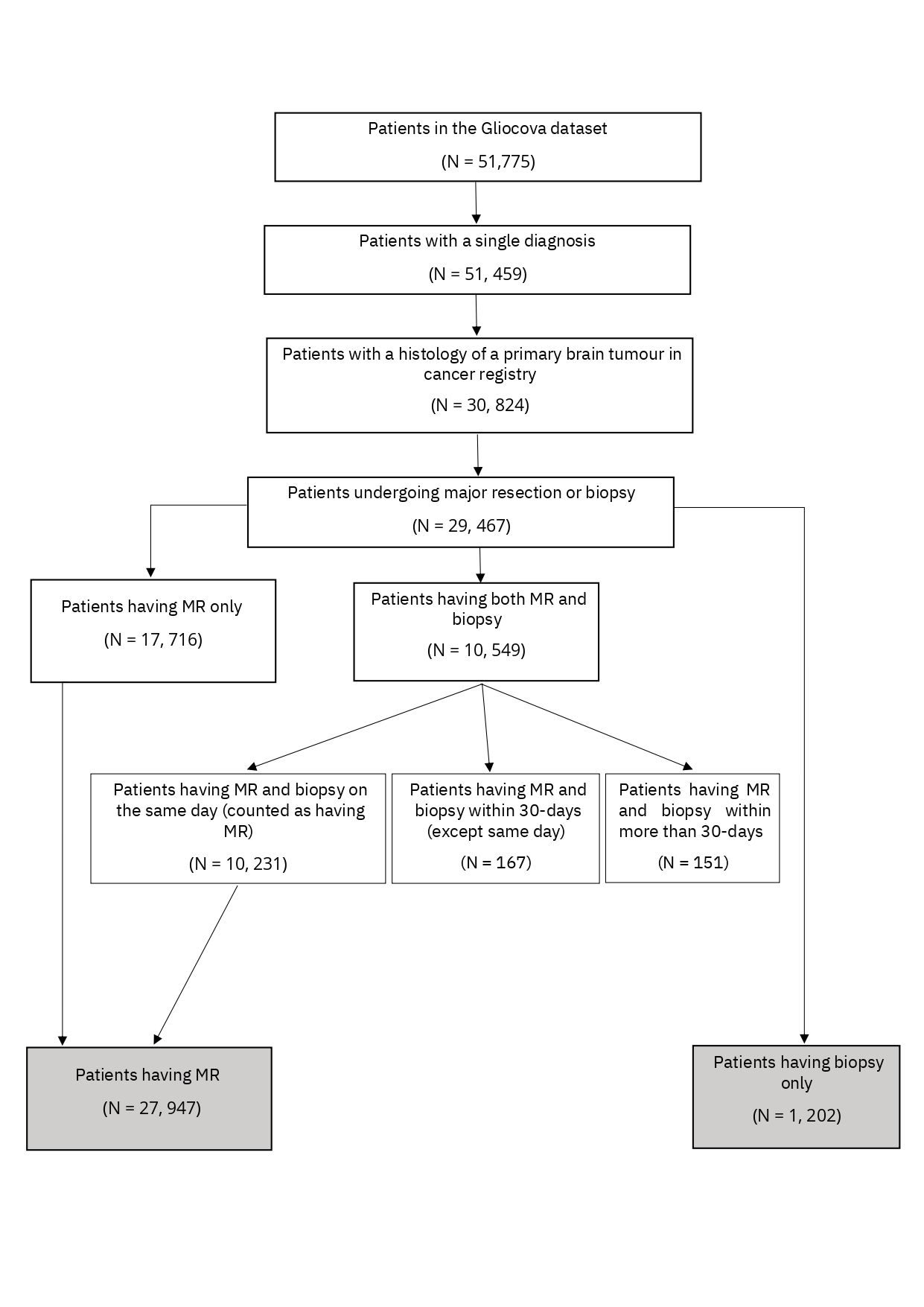

Supplement: S8 Fig — (DOCX) [file pone.0342011.s008.docx]
