# Supplementary material for: Estimating post-operative complication rates in patients with primary brain tumours from routine administrative data: A national cohort study
Source: PLoS One. 2026 Feb 19;21(2):e0342011. doi: 10.1371/journal.pone.0342011 (PMC12919839; doi:10.1371/journal.pone.0342011)
Supplement: S9 Table — (DOCX) [file pone.0342011.s009.docx]

**S9 Table. Distribution of the location of discharge for all patients undergoing first surgical intervention in the Gliocova dataset**

| **Destination of discharge** | **Number of patients** | **Percentage** |
| --- | --- | --- |
| **Usual place of residence** | 23294 | 80.3% |
| **Not applicable - Hospital Provider Spell not finished at episode end (i.e. not discharged) or current episode unfinished** | 2831 | 9.8% |
| **Hospital** | 2216 | 7.6% |
| **Patient died during the index admission** | 288 | 1.0% |
| **Care home/hospice** | 195 | 0.7% |
| **Other** | 178 | 0.6% |
| **Unknown** | 16 | 0.1% |
