# Supplementary material for: Estimating post-operative complication rates in patients with primary brain tumours from routine administrative data: A national cohort study
Source: PLoS One. 2026 Feb 19;21(2):e0342011. doi: 10.1371/journal.pone.0342011 (PMC12919839; doi:10.1371/journal.pone.0342011)
Supplement: S10 Table — (DOCX) [file pone.0342011.s010.docx]

**S10 Table. Most common ICD-10 diagnoses that could indicate reason of readmission**

| **Code** | **Description** | **Number of patients**  **(percentage of all readmitted patients)** |
| --- | --- | --- |
| **R51** | headache | 358(9.7%) |
| **Y83.6** | Surgical and other medical procedures as the cause of abnormal reaction, or of later complication, without mention of unintentional events at the time of the procedure: removal of other organ (partial/total) | 358(9.7%) |
| **R56.8** | other and unspecified convulsions | 336(9.1%) |
| **T81.4** | infection following a procedure, not elsewhere classified | 310(8.4%) |
| **G96.0** | CSF leak | 210(5.7%) |
| **R11** | nausea and vomiting | 188(5.1%) |
| **G97.8** | other post-procedural disorders of nervous system | 188(5.1%) |
