# Supplementary material for: Estimating post-operative complication rates in patients with primary brain tumours from routine administrative data: A national cohort study
Source: PLoS One. 2026 Feb 19;21(2):e0342011. doi: 10.1371/journal.pone.0342011 (PMC12919839; doi:10.1371/journal.pone.0342011)
Supplement: Attachment — Submitted filename: Response to reviewers.docx [file pone.0342011.s012.docx]

1. Please ensure that your manuscript meets PLOS ONE's style requirements, including those for file naming. The PLOS ONE style templates can be found at <https://journals.plos.org/plosone/s/file?id=wjVg/PLOSOne_formatting_sample_main_body.pdf> and <https://journals.plos.org/plosone/s/file?id=ba62/PLOSOne_formatting_sample_title_authors_affiliations.pdf>

Adjusted the title page and main text according to guidelines;

2. Please provide additional details regarding participant consent. In the ethics statement in the Methods and online submission information, please ensure that you have specified (1) whether consent was informed and (2) what type you obtained (for instance, written or verbal, and if verbal, how it was documented and witnessed). If your study included minors, state whether you obtained consent from parents or guardians. If the need for consent was waived by the ethics committee, please include this information.

If you are reporting a retrospective study of medical records or archived samples, please ensure that you have discussed whether all data were fully anonymized before you accessed them and/or whether the IRB or ethics committee waived the requirement for informed consent. If patients provided informed written consent to have data from their medical records used in research, please include this information.

Added details regarding consent to methods section (lines 192-203) as suggested and added also in the online submission;

3. Please provide a complete Data Availability Statement in the submission form, ensuring you include all necessary access information or a reason for why you are unable to make your data freely accessible. If your research concerns only data provided within your submission, please write "All data are in the manuscript and/or supporting information files" as your Data Availability Statement.

Updated the Data availability statement accordingly.

4. We note that you have indicated that there are restrictions to data sharing for this study. For studies involving human research participant data or other sensitive data, we encourage authors to share de-identified or anonymized data. However, when data cannot be publicly shared for ethical reasons, we allow authors to make their data sets available upon request. For information on unacceptable data access restrictions, please see <http://journals.plos.org/plosone/s/data-availability#loc-unacceptable-data-access-restrictions>.

Before we proceed with your manuscript, please address the following prompts:

a) If there are ethical or legal restrictions on sharing a de-identified data set, please explain them in detail (e.g., data contain potentially identifying or sensitive patient information, data are owned by a third-party organization, etc.) and who has imposed them (e.g., a Research Ethics Committee or Institutional Review Board, etc.). Please also provide contact information for a data access committee, ethics committee, or other institutional body to which data requests may be sent.

b) If there are no restrictions, please upload the minimal anonymized data set necessary to replicate your study findings to a stable, public repository and provide us with the relevant URLs, DOIs, or accession numbers. Please see <http://www.bmj.com/content/340/bmj.c181.long> for guidelines on how to de-identify and prepare clinical data for publication. For a list of recommended repositories, please see <https://journals.plos.org/plosone/s/recommended-repositories>. You also have the option of uploading the data as Supporting Information files, but we would recommend depositing data directly to a data repository if possible.

Please update your Data Availability statement in the submission form accordingly.

Updated the Data availability statement based on points 3 and 4 acordingly.

5. We note that the grant information you provided in the ‘Funding Information’ and ‘Financial Disclosure’ sections do not match.

When you resubmit, please ensure that you provide the correct grant numbers for the awards you received for your study in the ‘Funding Information’ section.

Added an updated funding disclosure to the cover letter as advised in the response letter;

In some cases we do not have specific grant numbers to provide thus these are not provided;

6. Thank you for stating the following in the Competing Interests section:
 Dr. Williams is employed by Imperial College Healthcare NHS Trust. He is also the medical director of PearBio (salary and share options).

Stephen J. Price is an advisor for TUMOURVUE Ltd (no financial involvement) and is the Chair of the Education Committee of the European Association for Neuro-oncology (EANO). He is on the speaker board for Medac GmbH and organises courses to train surgeons to use 5-ALA for which he is reimbursed, but has not run such a course since 2019.

Alex Bottle declared obtaining fees from AstraZeneca and Lilly outside the submitted work.

Please confirm that this does not alter your adherence to all PLOS ONE policies on sharing data and materials, by including the following statement: "This does not alter our adherence to PLOS ONE policies on sharing data and materials.” (as detailed online in our guide for authors <http://journals.plos.org/plosone/s/competing-interests>). If there are restrictions on sharing of data and/or materials, please state these. Please note that we cannot proceed with consideration of your article until this information has been declared.

Please include your updated Competing Interests statement in your cover letter; we will change the online submission form on your behalf.

Included the statement in the cover letter.

7. Please amend your list of authors on the manuscript to ensure that each author is linked to an affiliation. Authors’ affiliations should reflect the institution where the work was done (if authors moved subsequently, you can also list the new affiliation stating “current affiliation:….” as necessary).

Adjusted the affiliation list according to the submission guidelines. All authors already have at least 1 affiliation, some wanted to provide a few. Left only the primary affiliation on the submission form for each author and kept multiple affiliations if authors requested on the title page;

8. If the reviewer comments include a recommendation to cite specific previously published works, please review and evaluate these publications to determine whether they are relevant and should be cited. There is no requirement to cite these works unless the editor has indicated otherwise.


Reviewers' comments:

**Comments to the Author**
Review Comments to the Author

Reviewer #1: This is an important and timely study that addresses a clear gap in neurosurgical outcomes research: the absence of brain tumour–specific, scalable definitions of post-operative complications. Using a large national dataset, the authors develop and validate a novel complication list (ICL list), showing it identifies more patients with adverse outcomes than the OECD/PSI definitions. The methodology is rigorous and transparent, and the findings are of international relevance.

Major comments
 1. Clarity of aims and framing
 - The introduction is comprehensive but could be streamlined. The central research question (“Can we use administrative data to define and validate a broader, brain tumour–specific complication list at national scale?”) is somewhat diluted. A sharper aim statement at the end of the introduction would strengthen the framing. Made the introduction more concise.

2. Definition and validation of the ICL list
 - The Delphi process is described, but more detail is needed on how disagreements were resolved and how final codes were confirmed as complications. Were sensitivity analyses conducted excluding “borderline” diagnoses?

Added some information in the manuscript (lines 131-155 in the revised mansucript)

- It would be helpful to present the final ICL list (perhaps in the appendix) in a way that readers could apply in future work. Full list added as a separate table to manuscript as advised by other reviewers as well (Table 2, lines 228-229 in the revised manuscript)

3. Statistical modelling
 - The models are used only to validate the ICL list (not to predict outcomes). This is appropriate, but it should be more explicitly stated early to avoid reader confusion. Added this explicit statement in both methods and discussion section in the revision (lines 189-190 and lines 356-357)
 - The relatively modest C-statistics (<0.8) should be discussed further: what does this imply for future work on prognostication versus complication surveillance? Added sentence in discussion (lines 355-356; 364-374)

4. Interpretation of results
 The complication rate of 11.3% is convincing, but the clinical interpretation could be expanded. Which complications are driving this increase compared to OECD? Are they mainly infections, neurological events, systemic issues? This breakdown would aid clinical applicability. Added the description and the patient count for the most common complications from the ICL list to the manuscript (lines 269-273)

- The apparent paradox that meningioma patients have higher complication rates but lower mortality than GBM patients deserves more discussion. Added some discussion (lines 300-304)

5. Implications for practice and policy
 - The discussion could expand on how this work might be used in practice: e.g., benchmarking between centres, informing quality improvement, linking complication burden with health economic analyses. Added a sentence (lines 366-374)

- The authors should comment on whether the ICL list might be adaptable beyond England, or whether country-specific coding systems would require recalibration. Added a sentence (lines 369-370)

Minor comments
 - At times “OECD list,” “PSI,” and “OECD-defined complications” are used interchangeably; consistent language would improve clarity. Adjusted to be more consistent throughout the text;
 - ROC curves and cohort flowcharts are central to the argument — ensure they are high quality and easy to interpret. Updated figures and cohort flowchart to be of higher quality (Figs 1,2 and 3)
 - A few citations (e.g. [6], [39–41]) could be updated or clarified to ensure they align with the statements in text. Updated references;
 - Tighten the introduction to avoid repetition, and consider shortening the methods for clarity (details can be shifted to supplementary).

Tightened the introduction;

We have kept the methods section similar as to before revision, just including the additional details regarding Delphi process that were suggested by a few reviewers; We believe that the methods are already quite concise for this study and we already have a lot of supplementary material attached to it so we did not further move additional details to the supplementary material.

Recommendation
 The study is methodologically strong and of high clinical importance. Clarifying the aims, providing more transparency on the ICL list development, expanding the interpretation of findings, and sharpening the framing of implications will substantially strengthen the manuscript and broaden its impact.

Reviewer #2: This is a well-written and thoughtfully conducted study that provides new insight into postoperative complications following primary brain tumor surgery. By establishing a more comprehensive and specific list of complications, the authors enhance our ability to estimate and understand the true burden of postoperative morbidity in this patient group. The study contributes meaningfully to improving ethical and balanced discussions with patients and families, supporting more informed decision-making and risk communication.
 The analysis adds significant value to national data interpretation and future policy development. However, the study could be further strengthened by addressing how such findings might guide therapeutic decision-making or influence perioperative management strategies to mitigate risk. Overall, it represents an important step toward refining outcome measurement and advancing patient-centered neurosurgical care.

Added some discussion regarding the use and applicability of our findings 369-375

Reviewer #3: PONE-D-25-41552
 Estimating post-operative complication rates in patients with primary brain tumours from routine administrative data: A National Cohort study
 In this cohort study the authors analysed national administrative data from 29,018 adults undergoing brain tumour surgery in England to develop and validate an expanded definition of postoperative complications (the ICL list). They found that this definition identifies over five times more complications than current OECD indicators and conclude it enables meaningful estimation of neurosurgical complication rates at a national level.
 This is a very timely and well-designed study that addresses an important gap in neurosurgical outcomes research. The development of a scalable definition of postoperative complications using national administrative data is innovative and highly relevant for both clinical practice and health service evaluation.
 Comments
 The abstract for this kind of study should be presented in a structured format (Introduction, methods etc…) Added subsections in the abstract
 The introduction should be reworked: it is redundant and could be shortened, the aim should also be reported more clearly. Shortened the introduction
 The development of the ICL list is not described with sufficient clarity. The Authors should provide clearer criteria for how diagnoses were selected as complications and briefly explain the Delphi process used, including how consensus was reached. Added information to the manuscript (lines 131-155)
 The definition of the ICL complication list is central to the manuscript, but the full content of the ICL list only appears in supplementary material. The Authors should include either the full list or at a clearly structured summary table in the main text. Included the full list in the manuscript in Table 2 (lines 228-229)

The section titles that divide the discussion are not necessary and could be removed. Removed
 The Authors should better address the generalisability of their findings. Although the cohort is nationally representative within England, it is unclear how well the ICL complication list would apply to other healthcare systems with different coding practices or neurosurgical pathways. A brief discussion of this limitation is recommended. Added a sentence on this (lines 369-374)
 The conclusions section does not clearly summarise the main findings of the study. Instead, it focuses on future work without explicitly stating the key result: the ICL list identifies more postoperative complications than existing OECD indicators while maintaining model performance. The Authors should revise the conclusion to clearly state the study’s primary outcome. Phrases like “(by us and many others)” should also be avoided. Added a sentence on this. Deleted some additional information. Removed phrase that review objected to. (lines 377-380)

Reviewer #4: This is a well-executed national study addressing an important estimation of complication rates after brain tumour surgery using administrative data. The methodology, dataset, and statistical analysis are sound, and the manuscript is generally clear and well organized. The study’s use of the new Imperial College London (ICL) complication list is a meaningful advance, as it captures neurosurgery-specific complications missed by standard lists like the OECD’s. To improve clarity, the authors should better highlight what is novel about the ICL list in the introduction, summarizing how it was derived and validated. The methods section would benefit from a brief description of the main Delphi criteria used to select complications. The discussion should briefly address how this framework could be applied within NHS quality programs and its potential use in benchmarking or risk adjustment. Minor edits include shortening the abstract and defining “ICL list” at first mention, clarifying whether internal validation methods were used, and checking for minor errors. Overall, this is a strong and valuable contribution that merits publication after minor revisions for clarity and emphasis on the study’s novel aspects.

Defined ICL at first mention in the abstract and its novelty (lines 42-45)

Summarised how ICL list was derived and validated in the Introduction (lines 92-98)

Added an explanation in discussion regarding internal validation of the model (lines 362-364)

Shortened the abstract as requested;

Added further discussion regarding benchamrking (lines 372-374)

Reviewer #5: Summary
 In this paper the authors analyzed post-operative complications of adult brain tumor surgery on a large scale. They performed a retrospective cohort review of a national brain cancer database (GlioCova) in England between 2013-2018. The goal was to assess the rate of complications without individual chart review. First, they defined a novel list of brain tumor surgery complications (so-called ICL-list) with 51 diagnoses as coded by the ICD-10. They defined surrogate parameters to measure the rate of post-operative complications such as 30-day mortality (30-DM) and length of stay (LOS). They validated their ICL-list by running statistical models and comparing the performance to the list of Patient Safety Indicators (PSI) as developed by the US Agency for Health-care Research and Quality (AHRQ). As opposed to the novel ICL-list the PSI-list assess patient safety and is only defined by a very small number of key diagnoses. As a result, the authors analy-zed a cohort of 29,018 patients. The cohort was subdivided into age groups (18-40, 40-65 and 65+) and tumor type (glioblastoma / meningioma). They showed that the PSI-list captured a smaller proportion of patients with complications compared to the newly defined ICL-list and that both performed well in predicting 30-day mortality and length of stay. The novelty of their approach was to be able to use large scale healthcare data, rather than manually retrieved clinical chart data. The complication rate was 11.3%, the 30-day mortality was 2.3% and length of stay 5d and the 30-day readmission rate 12.7%. Older patients or with more co-morbidities, were more likely to have complications or die. They thoroughly discussed their findings in the light of the existing literature. In the future they plan to analyze the impact of complications on costs and adjuvant treatments.

Comments
 The authors are to be commended! To our knowledge their work is a novel approach to analyze complications of brain tumor surgery on such a large scale and to be able to extract data from routine health care databases. Interestingly, the extended ICL-list performs similar to the more restricted PSI-list in statistic models. The authors may further comment on this in the discussion part. Added a bit more on this in the discussion part (lines 355-366)

The manuscript is clearly written and needs no further language editing.

Please, briefly define and describe the ICL-list (novelty/ difference) in the abstract section. Added (lines 41-50)

Typos
 p. 2, l. 47 define “ ICL list”, Added (lines 42-45)
 p. 4, l. 101: typo “… be longer…”, Adjusted
 p 4., l.106: typo “… them. In this work…” Adjusted

Reviewer #6: I have read this manuscript with great interest. The authors address an important gap in neurosurgical quality assessment by developing a comprehensive complication list measurable at national level using administrative data. The study leverages an impressive dataset of nearly 30,000 patients with appropriate statistical methods. The manuscript is well-written and the research question clinically relevant. However, I have several concerns that need to be addressed before publication.

Major Concerns
 1. Validation of the ICL Complication List
 The validation strategy is insufficiently detailed. The Delphi process (S5 Figure) lacks critical information: What criteria did the five clinicians use to classify diagnoses as complications versus pre-existing conditions? How was consensus defined? What was inter-rater agreement?
 The validation against a "local dataset with access to detailed clinical notes" (lines 155-156) is too vague. How large was this dataset? How many of the 11 GlioCova-defined complications were verified as true complications? What proportion of coded diagnoses were confirmed when clinical notes were reviewed? Without this information, readers cannot assess whether the ICL list truly captures complications or includes pre-existing conditions.

The statistical validation—showing ICL models perform similarly to OECD models—is clever but indirect. It demonstrates that the expanded list includes diagnoses associated with worse outcomes, but does not prove all 51 diagnoses represent true complications. The authors should provide detailed validation data including inter-rater agreement from the Delphi process, and quantitative validation results from the local dataset, even if only for a subset of complications.

We cannot prove that all 51 diagnoses represent true complications due to the nature of the administrative data and the way it was collected - there is no way to distinguish whether the complication was present on admission or not.

Added additional details regarding the Delphi process (ines 131-155)

Edited the S5 figure to add more details as requested;

Added more information on the assessment from the local dataset (lines 150-153)

2. Coding Accuracy and Data Quality
 The limitations of administrative data are inadequately addressed. The authors mention that "some diagnoses might be miscoded or missed" (lines 331-332) without quantitative assessment. Several issues need attention:
 First, inter-center variability in coding practices is not examined, though this could lead to spurious variation in complication rates. Second, there is no discussion of financial incentives in the English NHS system—do hospitals receive additional payment for treating complications? Third, implications of coding accuracy (cited as ~80% in UK literature) for the estimated 11.3% complication rate are not discussed.
 Please add sensitivity analysis stratifying complication rates by center or region, if possible. Discuss financial coding incentives and their potential impact. Add a paragraph on how coding accuracy might affect findings, including direction and magnitude of potential bias.

Added comment regarding sensitivity analysis (lines 338-340)

Added discussion on coding accuracy, financial incentives (333-338)


3. Temporal Assignment of Complications
 How was it ensured that coded diagnoses represent post-operative complications rather than pre-existing conditions? ICD-10 coding in England does not consistently mark "present on admission." The authors excluded "chronic illnesses" (line 146), but many conditions could be either pre-existing or post-operative. For instance, headache is listed among common readmission diagnoses (S10 Table), but is also a common presenting symptom of brain tumors. This is particularly relevant for the 90.3% of complications from the GlioCova-defined list (line 216), which are not shown in the main manuscript.
 Please explicitly describe the strategy for ensuring diagnoses represent post-operative complications. Consider sensitivity analysis using only unambiguously post-operative complications (e.g., surgical site infection, post-operative hematoma). Present the 11 GlioCova-defined complications in the main text, not just in supplement.

We cannot prove that all 51 diagnoses represent true complications due to the nature of the administrative data and the way it was collected - there is no way to distinguish whether the complication was present on admission or not.

Added comments regarding post-operative complications and sensitivity analysis (lines 338-340)

Presented the complications in the main text, Table 2 (lines 229-230)

Minor Concerns
 4. Statistical Issues
 The dichotomization of LOS at 3 days (line 170) appears arbitrary—was this clinically or data-driven? The C-statistics are moderate (0.77 for mortality, 0.66 for LOS), limiting the strength of the validation approach. There is no mention of how missing data were handled.
 Justify the 3-day LOS cutoff. Clarify missing data handling. We removed patients that had missing LOS (lines 167-168). Cutoff was based on data distribution. Added additional details to the manuscript (lines 168-171)

5. Clinical Interpretation
 Table 2 shows a paradox: meningioma patients have higher complication rates than glioblastoma patients (e.g., 13.6% vs. 8.9% in age 18-40), yet much lower 30-day mortality (0.5% vs. 5.1%). Are meningioma complications less severe? Is this due to different surgical approaches or coding practices? This warrants discussion.
 Additionally, 30-day readmission is presented prominently but not used as an outcome in validation models. Why not? Discuss the meningioma-glioblastoma paradox. Consider including readmission in validation analyses.

Added some discussion of the meningioma vs glioblastoma paradox (lines 301-305)

Regarding including readmission in validation analyses, we have already included two outcome measures which are more directly affected by complication rate as readmission might be influenced by additional factors (e.g. variation in practice). The paper is already very long so we would be keen to not include an additional outcome measure.

6. Presentation Issues
 For a methods paper defining a new complication list, relegating the complete ICL list to supplement seems inappropriate. The ethics statement "N/A" is inadequate—at minimum, describe the data governance structure and institutional approval. Data availability states researchers must "contact us to enquire," but more clarity on the access process would aid reproducibility. Move at least the 11 GlioCova-defined complications to the main text. Provide proper ethics statement with data governance details. Clarify data access procedures.

Added ICL list to main text;

Added an ethics statement to the methods section; (192-203)

Clarified regarding data availability in the submission form;

Minor Editorial Points

Line 106: "we just want to be able to count them"—too colloquial, rephrase formally Removed when shortening the abstract
Line 200/Table 1: Age quintile notation confusing (1st-18, 2nd-46)—should be Q1=18, median=58, Q3=68 edited accordingly (lines 211-212)
 Table 1: "C71 Other" (16.5%) is very heterogeneous—provide more detail added more granular data to Table 1 (lines 216-217)
 Lines 222-225: LOS data with quintile notation is unreadable—use a table added a new table (Table 3, lines 238-240)
